# Complete Genome Sequences of Three Iberian *Brucella suis* Biovar 2 Strains Isolated from Wild Boars

**DOI:** 10.1128/genomeA.00618-14

**Published:** 2014-07-03

**Authors:** Ana Cristina Ferreira, Rogério Tenreiro, Maria Inácia Corrêa de Sá, Ricardo Dias

**Affiliations:** a Instituto Nacional de Investigação Agrária e Veterinária, IP, Lisboa, Portugal; b Universidade de Lisboa, Faculdade de Ciências, Centro de Biodiversidade, Genómica Integrativa e Funcional (BioFIG), Lisboa, Portugal

## Abstract

*Brucella suis* biovar 2 is the most common biovar isolated from wild boars (*Sus scrofa*) associated with transmission to outdoor-reared pigs in Europe. We report here the complete and annotated genome sequences of three strains isolated from wild boars in Portugal and Spain and belonging to the Iberian clone (haplotypes 2d and 2e).

## GENOME ANNOUNCEMENT

Bacteria from genus *Brucella* are Gram-negative *Alphaproteobacteria* that infect a wide range of mammals, including domestic mammals, many wild species, and humans. *Brucella suis* is one of the main pathogenic species worldwide that may cause abortion and infertility in its hosts, resulting in huge economic losses. *B. suis* is divided into five biovars, but only biovars 1, 2, and 3 are the etiological agents involved in swine brucellosis; *B. suis* biovar 2 is also found in wild boar (*Sus scrofa*), hares (*Lepus europaeus*) and biovar 3 in rodents (1). In Europe, biovar 2 is the most commonly isolated, and its prevalence in wild boars appears to be high throughout continental Europe, implicating them as a potential source of transmission to outdoor-reared pigs (1–3). *B. suis* biovar 2 strains PT09143 (haplotype 2e), PT09172 (haplotype 2d), and Bs143CITA (haplotype 2e) were isolated from wild boars, biotyped according to standard bacteriological procedures (4), and characterized through PCR-restriction fragment length polymorphism (RFLP) analysis for the *omp2a*, *omp2b*, and *omp31* genes and a multilocus variable-number tandem-repeat (VNTR) assay (MLVA). All strains presented specific patterns of Iberian isolates, being distinct from other biovar 2 strains isolated in central European countries. Here, we report the complete and annotated genome sequences of these three strains. Genomic libraries were created using the TruSeq DNA sample preparation kit. The genomic sequences were obtained by Illumina HiSeq 2000 technology, with a paired-end 35-bp protocol generating a total of 8,408,102, 12,172,794, and 10,795,456 high-quality reads (Phred score, >30) for PT09143, PT09172, and Bs143CITA, respectively. The reads were *de novo* assembled using the de Bruijn graph method (Velvet version 1.2.09) (5), and 116 (*N*_50_, 56,902 bp), 65 (*N*_50_, 100,353 bp), and 61 (*N*_50_, 122,343 bp) contigs were generated for PT09143, PT09172, and Bs143CITA, respectively. Visual inspection of the alignment of fastq reads for each contig was performed using Tablet 1.14.04.10. Scaffolding was guided by an optical mapping method (MapSolver version 3.2; OpGen Technologies, Inc.), and gap filling was performed by PCR and the Sanger method (6). Low-coverage (<20×) regions and all indels (compared with *B. suis* ATCC 23445) were confirmed by Sanger resequencing. A total of 3,378 (PT09143), 3,380 (PT09172), and 3,375 (Bs143CITA) coding DNA sequences (CDSs) were predicted through RAST (7). Three copies of 5S, 16S, and 23S rRNA genes were identified using RNAmmer (8), and a set of 54 copies of tRNA genes were predicted with tRNAscan-SE 1.21 (9). The three genomes have similar sizes and are composed of two circular chromosomes with approximately 1.93 and 1.40 Mb and an overall G+C content of 57.2%. The genome sequences of PT09143, PT09172, and Bs143CITA presented 72% and 71% similarity compared with the reference genomes of *B. suis* ATCC 23445 (biovar 2, accession no. CP000911 and CP000912) and *B. suis* 1330 (biovar 1, accession no. AE014291 and AE014292), respectively. This relative low level of similarity is due to the presence of a 944-kb inversion in chromosome I in all three strains in comparison to both reference strains.

### Nucleotide sequence accession numbers.

The complete genome sequences have been deposited in GenBank under accession no. CP007691/CP007692, CP007693/CP007694, and CP007695/CP007696 for *B. suis* PT09143, PT09172, and Bs143CITA chromosomes I/II, respectively.
